# Increased serum resistin level is associated with coronary heart disease

**DOI:** 10.18632/oncotarget.15707

**Published:** 2017-02-25

**Authors:** Jing-Zhan Zhang, Ying Gao, Ying-Ying Zheng, Fen Liu, Yi-Ning Yang, Xiao-Mei Li, Xiang Ma, Yi-Tong Ma, Xiang Xie

**Affiliations:** ^1^ Department of Cardiology, First Affiliated Hospital of Xinjiang Medical University, Urumqi, China; ^2^ Department of Dermatology, People's Hospital of Xinjiang Uygur Autonomous Region, Urumqi, China; ^3^ Department of Cadre Ward, First Affiliated Hospital of Xinjiang Medical University, Urumqi, China

**Keywords:** coronary heart disease, serum resistin, meta-analysis

## Abstract

To explore the relationship between the serum resistin level and different types of coronary heart diseases (CHD). Literature was retrieved by formal searching of PubMed, Web of Science, Google Scholar, the Cochrane Library, Wanfang Data, China Biological Medicine Database (SinoMed) and China National Knowledge Infrastructure (CNKI) and by hand searching of reference lists of related articles. RevMan5.3 statistical software was utilized for processing and analysis. A total of 22 literatures involving 2070 subjects were included. Meta-analysis showed that the level of serum resistin in the patients with stable angina (SA), unstable angina(UA) or acute myocardial infarction (AMI) were significantly higher than those of normal controls, respectively [SMD(95% CI) were 1.97(1.15, 2.79), 2.54(1.76, 3.31), and 3.62(2.62, 4.62), all *P*<0.00001]. Serum resistin level in patients with UA or AMI was higher than those in patients with SA, respectively [SMD=0.90, 95CI(0.28,1.52), *P*=0.005], [SMD=2.28, 95%CI(0.74, 3.82), *P*=0.004]. The level of serum resistin in patients with AMI was also higher than those in patients with UA [SMD=1.22, 95%CI(0.58, 1.85), *P*=0.0002]. The study demonstrated that increased serum resistin level is significantly associated with the severity of CHD.

## INTRODUCTION

Coronary heart disease (CHD) is the occurrence of coronary atherosclerosis events that causes the stenosis or obstruction of the vessel lumen and leading to cardiac ischemia, anoxia or necrosis. Chronic inflammation is considered to be a risk factor for increasing the risk of coronary events, which can make atherosclerotic plaques in coronary artery prone to rupture [1, 2]. Resistin is a newly discovered adipocyte factor. Since the discovery of resistin, many studies mainly focused on the relationship among it with insulin resistance and obesity, and suggested that resistin may be an important factor which can lead to obesity, insulin resistance and type 2 diabetes mellitus (T2DM).

In recent years, some studies show that resistin is related to many inflammatory markers and may be involved in the atherosclerosis, which plays an important role in the development of CHD. The relationship between serum resistin level and the severity of CHD has attracted significant clinical research interest. Therefore, we conducted a meta-analysis of the existing published studies on this topic to evaluate the strength of the association between serum resistin level and CHD.

## RESULTS

### Study characteristics

**Figure 1:**
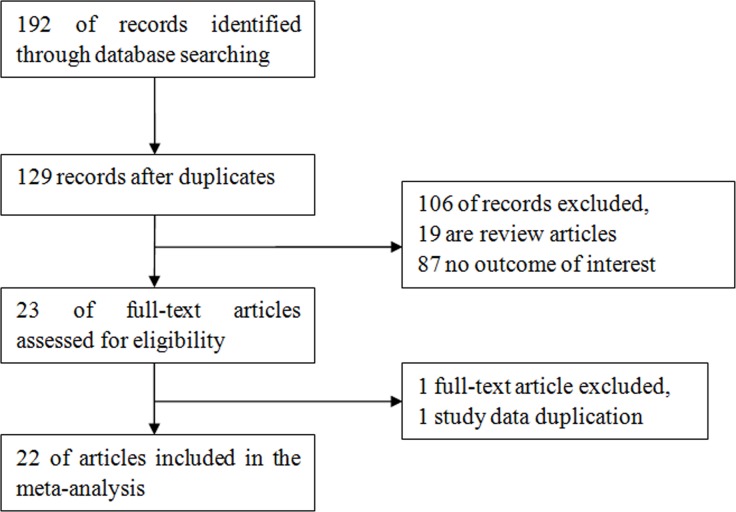
Flow diagram of study identification

We retrieved a total of 192 studies. After duplicates removed, only 129 full-text studies were evaluated. After exclusion of review articles, a total of 22 studies [3–24] were included in the final meta-analysis according to the inclusion criteria. There are 2070 subjects, including 648 control subjects and 1422 CHD patients (447 cases in SA group, 447 cases in UA group, 528 cases in AMI group). The NOS results showed that the methodological quality was generally high. Figure 1 shows the process of literature selection. Table 1 showed the characteristics of included studies.

**Table 1:** Characteristics of the studies reporting the serum resistin level and different types of CHD

First author	Publication year	Country	Detection Assay	Normal control group			SA group			UA group			AMI group		
				Mean	SD	N	Mean	SD	N	Mean	SD	N	Mean	SD	N
Cao[3]	2009	China	ELISA	3.17	0.35	40				3.7	0.59	52	4.49	0.76	50
Chu[4]	2008	China	ELISA	4.17	2.24	26				7.34	3.44	19	15.12	5.06	20
Geng[5]	2013	China	ELISA	0.57	0.08	30				0.96	0.11	30	1	0.11	30
Guo[6]	2008	China	ELISA	0.22	0.15	15	1.99	0.83	31	3.46	0.99	23	4.76	0.85	19
Huang[7]	2006	China	ELISA	8.92	7.8	24	16.18	7.8	24	18.78	8.56	12	26.74	8.7	28
Erer[8]	2013	Turkey	ELISA	2	1.05	33							3.71	4.2	132
Korah[9]	2011	Egypt	ELISA	5.47	0.81	15							13.59	3.6	40
Li[10]	2006	China	ELISA	4.17	2.24	26				7.67	4.28	18	15.12	5.06	19
Li[11]	2011	China	ELISA	0.13	0.28	35	0.14	0.13	35	0.18	0.23	69			
Liu[12]	2004	China	ELISA	3.2	0.48	30	5.68	1.49	22	5.79	1.52	20	5.86	1.67	18
Liu[13]	2010	China	ELISA	1.09	0.35	8	2.41	0.67	12	4.87	1.72	28			
Ma[14]	2008	China	ELISA				2.1	0.7	40				4.1	0.8	37
Qiao[15]	2007	China	ELISA				3.45	0.56	22	5.59	0.75	19	8.16	0.79	24
Sang[16]	2009	China	ELISA	6.94	0.9	20				10.42	0.7	18	12.49	0.88	18
Sinan[17]	2014	Turkey	ELISA	0.017	0.0085	50	0.02539	0.0134	80	0.025	0.018	39			
Tang[18]	2011	China	ELISA	7.4	2.1	40									
Wang[19]	2009	China	ELISA	0.49	0.4	67	0.66	0.4	70						
Wang[20]	2010	China	ELISA	3.17	0.15	30	5.41	0.27	18	5.61	0.28	20	5.65	0.33	22
Wang[21]	2012	China	ELISA	6.94	0.9	20				10.42	0.7	25			
Wang[22]	2012	China	ELISA	44.68	6.89	25				49.23	7.81	25	54.17	8.57	25
Yang[23]	2011	China	ELISA	0.262	0.1119	90	0.5183	0.1284	93						
Zhang[24]	2009	China	ELISA	12.16	2.64	24				19.16	1.18	30	23.46	1.49	46

CHD Coronary heart disease, SA stable angina, UA unstable angina, AMI acute myocardial infarction, SD standard deviation, N sample number, ELISA Enzymelinked Immunosorbent Assay

### Main results and heterogeneity

#### Association of resistin level with SA

**Figure 2:**
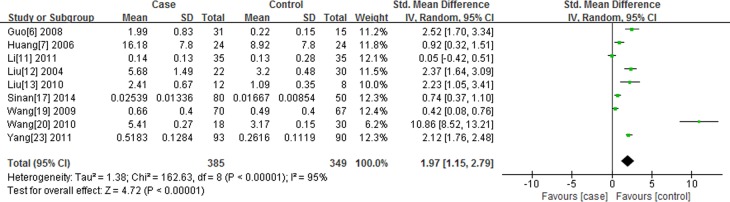
Forest plot of the serum resistin level between the study of SA and the normal control group, the horizontal lines correspond to the study-specific SMD and 95% CI, respectively The area of the squares reflects the study-specific weight. The diamond represents the pooled results of SMD and 95%CI.

There are 9 studies reporting the association of resistin level with SA. I^2^ test indicated that the heterogeneity is significant (*P* < 0.00001, I^2^ = 95.0%), therefore, the random-effects model (DerSimonian and Laird) was applied to perform meta-analysis. We found serum resistin level in SA group is higher than that in normal control group [standard mean difference (SMD) = 1.97, 95%CI: 1.15-2.79, *P* < 0.00001] (Figure 2).

#### Association of resistin level with UA

There are 15 studies reporting the association of resistin level with UA. I^2^ test indicated that the heterogeneity is significant (*P* < 0.00001, I^2^ = 95.0%), therefore, the random-effects model (DerSimonian and Laird) was applied to perform meta-analysis. Serum resistin level of UA is higher than that in normal control group [SMD = 2.54, 95%CI: 1.76-3.31, *P* < 0.00001] (Figure not shown).

#### Association of resistin level with AMI

There are 13 studies reporting the association of resistin level with AMI. I^2^ test indicated that the heterogeneity is significant (*P* < 0.00001, I^2^ = 95.0%), therefore, the random-effects model (DerSimonian and Laird) was applied to perform meta-analysis. The results show that AMI serum resistin level is higher than that in normal control group [SMD (95%CI) = 3.62 (2.62, 4.62), *P* < 0.00001] (Figure not shown).

### Comparison of UA with SA in serum resistin level

**Figure 3:**
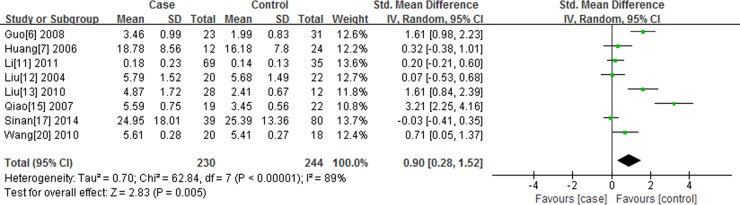
Forest plot of the serum resistin level between the study of the UA group and SA group, the horizontal lines correspond to the study-specific SMD and 95% CI, respectively The area of the squares reflects the study-specific weight. The diamond represents the pooled results of SMD and 95%CI.

There are 8 studies reporting the association of resistin level with UA compared to SA. I^2^ test indicated that the heterogeneity is significant (*P* < 0.00001, I^2^ = 89.0%), therefore, the random-effects model (DerSimonian and Laird) was applied to perform meta-analysis. The results show that serum resistin level in UA group is higher than that in SA group [SMD (95%CI) = 0.90 (0.28, 1.52), *P* < 0.05] (Figure 3).

### Comparison of AMI with SA in serum resistin level

There are 5 studies reporting the association of resistin level with AMI compared to SA. I^2^ test indicated that the heterogeneity is significant (*P* < 0.00001, I^2^ = 95.0%), therefore, the random-effects model (DerSimonian and Laird) was applied to perform meta-analysis. The results show that AMI serum resistin level is higher than SA group [SMD (95%CI) = 2.28 (0.74,3.82), *P* = 0.005].

### Comparison of AMI with UA in serum resistin level

There are 12 studies reporting the association of resistin level with AMI compared to UA. I^2^ test indicated that the heterogeneity is significant (*P* < 0.00001, I^2^ = 92.0%), therefore, the random-effects model (DerSimonian and Laird) was applied to perform meta-analysis. The results show that AMI serum resistin level is higher than UA group [SMD (95%CI) = 1.22 (0.58,1.85), *P* = 0.0002].

### Sensitivity analysis

The contribution of each study to the pooled estimate was performed in order to assess the sensitivity analyses. We excluded one individual study at a time and recalculated the pooled *P* or OR. All studies did not substantially change the pooled point estimate, which indicated the reliability of our results.

### Publication bias

**Figure 4:**
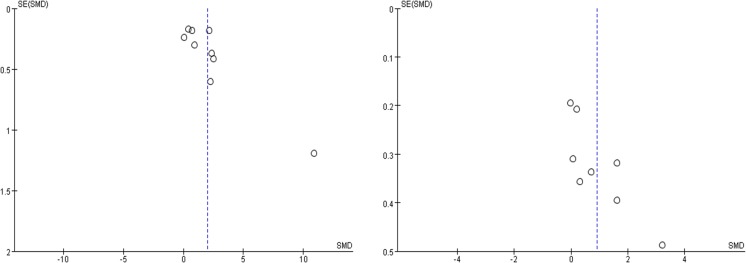
**A. Funnel plot for publication bias tests about the serum resistin level between the study of SA and the normal control group. B**. Funnel plot for publication bias tests about the serum resistin level between the study of the UA group and SA group. Each point represents a separate study for the indicated association. The *horizontal and vertical axis* correspond to the SMD and confidence limits. *SMD standardized mean differences, SE standard error*.

The publication bias was evaluated using the funnel plot. The funnel plot of each study is basically symmetrical. No visual publication bias was found, as shown in Figure 4.

## DISCUSSION

The present meta-analysis was conducted by pooling both ORs and SMD and found that serum resistin levels may be an independent risk of CHD. It may be the first attempt to synthesize the existing studies to evaluate the association of serum resistin levels and CHD.

Resistin is secreted by white adipose tissue. It is a cysteine-rich protein which belongs to a family of cysteine-rich secretory proteins called resistinlike molecules (RELMs) [25]. Human resistin gene is located on chromosome 19, including four exons and three introns [26].

Elevated resistin levels were found in acute inflammation, accompany with several certain inflammatory factors [27] such as C-reactive protein, interleukin-6, and tumor necrosis factor. There are several studies about resistin levels and obesity-related cancer risk [28]. Many studies have shown that the resistance elements can be used as a marker of myocardial injury, diagnosis and prognosis of CHD. Verma et al [29] found that resistin activates human endothelial cells (ECs) *in vitro*; when incubated with ECs, recombinant resistin increases endothelin-1mRNA expression and protein secretion, also increases vascular cell adhesion molecule-1 and monocyte chemoattractant protein-1 expression, also impairs tumor necrosis factor receptor-associated factor-3 expression. These activities support the hypothesis that resistin and cardiovascular disease. A previous study reported by Lee et al [30] showed that resistin was an independent predictor of cardiovascular major adverse events in AMI patients. Yang et al. [23] found that plasma resistin may promote the formation of vulnerable plaque. Plasma resistin levels were significantly increased in CHD patients, especially in ACS, and gradually increased with the severity of CHD. Sinan 2014 [17] and Liu 2004 [12] found that increased serum resistin level is a predictor of CHD and there was a positive correlation between the Gensini score and the serum resistin level. The Gensini score is a parameter used to assess the prevalence and severity of CAD [31]. However, the relationship between increased serum resistin levels and pathogenic severity of CHD was not reported. In the present study, we conducted a meta-analysis of the published studies in order to evaluate the strength of the association between serum resistin level and different types of CHD.

The results showed that the standard deviation of resistin levels in SA, UA and AMI groups were higher compared to normal controls. Our Meta-analysis also found that the standard deviation of resistin levels in AMI patients is higher than UA and SA group, and UA group serum resistin levels is higher than the SA group. The result indicated that serum resistin levels gradually increased in SA group, UA group and AMI group, which suggested that serum resistin level and severity of CHD are closely related.

In addition, Erer HB [8] reported that serum resistin levels were similar in patients with STEMI and NSTEMI. Canga et al. [32] demonstrated a significant increase of serum resistin levels in patients with slow coronary flow compared to patients with normal coronary flow. Reilly et al reported that serum resistin level was independently associated with coronary artery calcification.

Heterogeneity is a potential problem that may affect the interpretation of the results. Heterogeneity may be attribute to the potential confounding resulted from diversity in sample-sizes, age, design, difference of testing instruments, severity of CHD, experimental method, and the interaction with other risk factor. To better interpret the results, other limitations of our meta-analysis should also be acknowledged. For one thing, the relative paucity of quality data and some inevitable publication bias may exist in our results. Only full text articles published in English and Chinese were included in this meta-analysis. Thus some eligible studies, which were unpublished or reported in other languages, were likely missed. Cultural background factors can also affect the decision to publish, making researchers more or less likely to report or edit negative results in some areas of research. For another, the characteristics of subjects, treatment plan and the difference of sampling time may have some effect on the results of the study. Furthermore, the difference between the reagents and the measuring instruments used in the research is the main reason of the heterogeneity of the study. We did not have access to enough data to make a subgroup analysis, this may affect the representative of our meta-analysis.

Despite these limitations or disadvantages, our meta-analysis did have some advantages. First, this is the first meta-analysis that consolidates the available information to explore the relationship between the serum resistin level and different types of CHD. A systematic review of the association between the serum resistin level and different types of CHD is able to overcome the limitation of the small sample sizes of the study populations by increasing the sample size, thus generating more robust data. Second, the quality of the case-control studies included in our meta-analysis was satisfactory and met our inclusion criteria.

In conclusion, serum resistin levels gradually increased with the severity of coronary heart disease, suggesting clinical serum resistin levels according to changes in the development of coronary heart disease can determine the extent of disease, disease forecasting, dynamic monitoring of serum resistin levels on the treatment and prognosis of coronary heart disease has clinical significance, reduce serum resistin levels of coronary heart disease is expected to become a new therapeutic target.

## MATERIALS AND METHODS

### Literature search

We searched literatures describing the association between resistin and different types of CHD, including acute myocardial infarction (AMI), stable angina (SA) and unstable angina (UA). Literature was retrieved by formal searching of electronic databases (PubMed, Web of Science, Google Scholar, the Cochrane Library, Wanfang Data, SinoMed and CNKI) and by hand searching of reference lists of related articles. These computer searches were limited to English and Chinese language articles before May 2015. The following keywords were used for searching: “acute myocardial infarction” OR “AMI” OR “stable angina” OR “SA” OR “unstable angina” OR “UA” OR “coronary heart disease” OR “CHD” OR “coronary artery disease” OR “CAD” OR “Acute coronary syndrome” OR “ACS” AND “serum resistin”.

### Selection criteria

The diagnosis and severity of CHD was fitted to the examination results of coronary arteriography, clinical symptoms combined with echocardiography, treadmill exercise test, electrocardiogram and myocardial perfusion imaging in Emission Computed Tomography.

The inclusion criteria were as follows: (1) Published literatures related to the association of serum resistin level with CHD, including stable angina (SA), unstable angina (UA) and acute myocardial infarction (AMI); (2) Independent case-control studies using either a hospital-based or a population-based design; (3) The original studies must provide the number of each group and the mean and standard of serum resistin.

#### Excluded criteria

(1) Duplicated data; (2) the original data could not be extracted.

### Data extraction

Two authors (JZ Zhang and Y Gao) independently extracted the original data. Disagreement was resolved by discussion. If the two authors could not reach a consensus, the result was reviewed by a third author (X Xie). The extracted data were consisted of the follow items: the first author's name, publication year, population(Ethnicity), methods, study design, matching criteria, sex, total number of cases and controls, and age (years).

### Quality assessment

Study quality was assessed by the Newcastle-Ottaw scale [33], which uses a “star” rating system to judge the quality of all observational studies. The NOS ranges between zero (worst) up to nine stars (best). Studies with a score equal to or higher than seven were considered to be of high quality. Two investigators (Y Gao and YY Zheng) independently assessed the quality of the included studies, and the results were reviewed by a third investigator (YT Ma). Disagreement was resolved by discussion.

### Statistical analysis

We utilized Review Manager 5.33 (Cochrane Collaboration, The Nordic Cochrane Centre, Copenhagen) and Stata12.0 software to perform the meta-analysis in the present study. Heterogeneity among studies was assessed by I^2^ statistic, *P* < 0.10 and I^2^ > 50% indicated evidence of heterogeneity [34]. If heterogeneity existed among the studies, the random effects model [35] was used to estimate the pooled SMD. Otherwise, the fixed effects model [36] was adopted. The standard mean difference (SMD) and corresponding 95% confidence interval (CI) was utilized to assess the associations. The potential publication bias was investigated using a funnel plot. Egger's test (*P* < 0.05) was also considered to be representative of statistically significant publication bias [37], which was conducted with the Stata12.0 software.
